# Quality control of cervical cytology using a 3-type HPV mRNA test increases screening program sensitivity of cervical intraepithelial neoplasia grade 2+ in young Norwegian women—A cohort study

**DOI:** 10.1371/journal.pone.0221546

**Published:** 2019-11-05

**Authors:** Bjørn Westre, Anita Giske, Hilde Guttormsen, Sveinung Wergeland Sørbye, Finn Egil Skjeldestad

**Affiliations:** 1 Department of Pathology, Ålesund Hospital, Møre and Romsdal Health Trust, Ålesund, Norway; 2 Department of Clinical Pathology, University Hospital of North Norway, Tromsø, Norway; 3 Research Group Epidemiology of Chronic Diseases, Department of Community Medicine, UiT The Arctic University of Norway, Tromsø, Norway; Azienda Ospedaliero Universitaria Ospedali Riuniti di Ancona Umberto I G M Lancisi G Salesi, ITALY

## Abstract

Within 2021, Norway intends to complete implementation of HPV DNA-based primary screening for cervical cancer for women 34–69 years, while continue cytology-based screening for women 25–33 years. Over the recent years, the incidence of cervical cancer has increased by 30% among women younger than 40 years. In this subset of women, nearly 30% were diagnosed with a normal smear, as most recent smear, prior the cancer diagnosis. This observation demands quality control of normal smears. The aim of this study was to assess increase in program sensitivity of CIN2+ after follow-up of women with false negative Pap-smears testing positive for a 3-type (-16, -18, -45) HPV mRNA test in a cohort design over one screening interval. 521 women, aged 23–39 years, and no prior history of CIN1+ or HSIL, with an ASC-US or worse smear (ASC-US+) and 1444 women with normal screening cytology comprised the study cohorts. The positivity rate for the 3-type HPV mRNA was 1.9% (28/1444). Rescreening revealed 23 women with ASC-US, two women with LSIL, two women with ASC-H, and one woman with AGUS. If the HPV mRNA-positivity rate and histology findings from samples rescreened were applied to all women with normal cytology, an estimated increase in screening sensitivity of 16.4% (95% CI:15.3–17.5) for CIN2+ and 17.3% (95% CI:16.2–18.4) for CIN3+ were achieved. By rescreening less than 2% of women with normal cytology positive for a 3-type HPV mRNA test, we achieved a significant increase in screening program sensitivity.

## Introduction

Cytology screening for cervical cancer has been effective in reducing cervical cancer incidence in countries with high-coverage and high-quality screening programs [1–4]. In the Nordic countries, this applies to slow growing cancers among women above 40 years of age, but not to women younger than 40 years [3,5,6].

Primary HPV-screening with DNA-based tests find high-grade cervical intraepithelial neoplasia grade 2 and 3 (CIN2+) at an earlier time point than cytology based screening do [7]. However, over two screening rounds there were no difference in detection rates of CIN2+ [8]. A meta-analysis of randomized screening trials against cervical cancer reported more invasive cervical cancer cases detected in the cytology-arm compared with the HPV-arm [9]. In women younger than 30 years primary HPV DNA based screening is very ineffective and not cost-effective due to the high prevalence of transient HPV-infections [10–12].

Worldwide HPV 16 and 18 are the most prevalent oncogenic types detected from cervical cancer tissue [13–15]. Cancers associated with HPV-16/-18/-45 are more often diagnosed at younger ages, supporting the hypothesis of faster progression to cancer than precancerous lesions associated with other HPV types [16]. These three types may account for 98% of all HPV-positive cervical adenocarcinomas diagnosed in younger women [16–18].

In Norway, there has been 30% increase in cervical cancer in women younger than 40 years over the past 10 years [19]. About half of Norwegian women diagnosed with cervical cancer have attended screening, of whom more than 57% had “normal” cytology at the most recent smear less than 4 years before the cancer diagnosis. For the years 2007 through 2016, this comprised 344 women less than 40 years with a delayed diagnosis of cervical cancer (Table 1). Rescreening of “normal” smears in women with cervical cancer often reveal abnormal cells overlooked or misinterpreted in previous screening rounds [20,21].

**Table 1:** Number of cervical cancers among women < 70, < 25, and < 40 yrs., number of women < 40 yrs. with smears within 4 years of cancer diagnosis, and proportion (%) of women with normal last smear before start of cascade of smears leading to a cancer diagnosis, Norway, 2007 through 2016 and total.

Year	No. of cervical cancers < 70 yrs.	No. of cervical cancers < 25 yrs.	No. of cervical cancers 25–39 yrs.	No. of women < 40 yrs. with smears < 4 yrs. of cancer diagnosis	% women < 40 yrs. with normal last smear before diagnosis of cancer
2007	206	2	64	43	48.8
2008	243	5	93	65	55.4
2009	260	6	94	62	46.7
2010	278	4	106	67	65.7
2011	259	5	107	66	51.5
2012	278	1	100	56	48.2
2013	243	6	74	40	55.0
2014	306	6	133	75	56.0
2015	338	5	126	67	58.0
2016	301	13	108	62	53.2
Total	2 712	53	1 005	603	57.0

Data sources: Annual reports The NCCSP 2008 through 2017, Institute of Population based Cancer Research. Oslo. Norway (in Norwegian).

In the present study, we assess the impact of quality control by co-testing cytology normal young women with an HPV mRNA test targeting HPV-16/-18/-45 on screening program sensitivity, and evaluate the subsequent workload for the screeners caused by re-screening smears from women who had a positive mRNA test.

## Material and methods

The Department of Pathology, Ålesund Hospital, assesses approximately 12 000 cervical smears annually. The department uses the Bethesda system for classification of cervical cytology and the WHO histological classification of tumors in cervical biopsies (http://screening.iarc.fr/colpochap.php?chap=2). One experienced pathologist (BW) reviewed all biopsies. Biopsies with uncertain cellular changes were immunostained with p16 (INK4a) (Roche mtm laboratories AG). If there was a discrepancy between biopsy and treatment histology, the most severe histology determined the endpoint.

HPV mRNA were detected using PreTect SEE (PreTect AS, Klokkarstua, Norway) a diagnostic kit for the qualitative detection and direct typing of E6/E7 mRNA from HPV 16, 18 and 45 according manufacturer’s instructions. The kit contains an intrinsic sample control (ISC) targeting a human housekeeping gene to assess specimen quality and reveal possible factors that may inhibit amplification. PreTect SEE sequences correspond to a subset of genotypes in PreTect HPV-Proofer (PreTect AS) and make use of real-time NASBA technology, an enzymatic one-step amplification process able to amplify RNA under isothermal conditions (41°C).

Following national guidelines in delayed triage, Ålesund Hospital utilized the HPV DNA test Cobas 4800 (Roche Molecular Diagnostics), which detects 14 HPV types. In addition, HPV mRNA was detected using PreTect HPV-Proofer (PreTect AS, Norway), which detects E6/E7 mRNA from types-16, -18, -31, -33, -45.

During the study period, the Norwegian Cervical Cancer Screening Programme (NCCSP) recommended delayed triage of women with minor cervical lesions with repeat cytology and HPV testing 6–12 months after the index diagnosis of atypical cells of undetermined significance (ASC-US) or low-grade squamous intraepithelial lesion (LSIL). In order to increase sample size (power) without compromising validity of testing, and to mimic the real life of follow-up in the NCCSP, we expanded the triage follow-up window from 90 to 540 days after the index smear.

Referred to colposcopy/biopsy were women with high-grade squamous intraepithelial lesions (HSIL) or repeated ASC-US/LSIL with a positive HPV test. Women with a normal smear and a positive HPV test were recommended a repeat HPV test within 12 months, whereas women with an ASC-US/LSIL/normal smear with a negative HPV test returned to regular screening at a three-year interval [22].

We sampled cells for conventional cytology (Pap smear) with a wooden spatula and a brush from the cervix uteri, and placed the material directly onto a glass slide followed by fixation. The same brush was rinsed in ThinPrep (Cytec Corporation, Marlborough, USA), and sent for subsequent HPV mRNA testing. In Norway, many hospitals have switched from conventional Pap smears to liquid-based cytology (LBC), but Ålesund Hospital still used conventional Pap smears over the study years.

From April 4^th^, 2013, the department started rescreening all normal smears among women aged 23–39 years with a concurrent positive HPV mRNA test. During the inclusion-window 4747 women, 23 through 39 years of age had a valid smear. After exclusion of women with a history of CIN1+ (n = 339) or HSIL without CIN (n = 42), the study population comprised 4366 women. During primary screening we defined four follow-up cohorts; a passive arm of normal smears not tested for HPV (control arm), normal smears testing positive with the 3-type mRNA test (mRNA positive arm), follow-up of ASC_US-LSIL as the ASC_US-LSIL arm, and the HSIL-arm as follow-up of women with HSIL. Follow-up continued for one screening interval through December 31, 2017.

All analyses were done in SPSS, version 24.0, as differences between means (detection rates) at significance level p < 0.05.

The Regional Committee for Medical and Health Research Ethics (REC Central) has approved the protocol as a quality assurance study in laboratory work (2014/669/REK midt). Norwegian regulations exempt quality assurance studies from written informed consent from the patients.

## Results

In this low risk population of women younger than 40 years index smear were normal in 88.1% (3845/4366) of the women, 8.5% (370/4366) had ASC-US, 1.9% (82/4366) had LSIL (ASC-US/LSIL-arm), and 1.6% (69/3466) had HSIL, ASC-H, AGUS or ACIS (HSIL-arm). In samples diagnosed as normal, 1444 of 3851 (37.5%) had liquid based cytology (LBC) available for HPV-testing. In this subset of women, the positivity rate for HPV mRNA was 1.9% (28/1444) (mRNA-positive arm). Rescreening samples revealed 23 women with ASC-US, two women with LSIL, two women with ASC-H and one woman with AGUS. Finally, the control arm comprised 2401 women not tested for mRNA HPV (3845–1444).

At triage, eight of 28 women in the mRNA-positive arm and 199 out 452 women in the ASC-US/LSIL arm returned to screening after having a negative HPV DNA test result and a normal, ASC-US, or LSIL cytology reading. In the ASC-US/LSIL arm, 35 women had no cytology follow-up, whereas 18 women had incomplete cytology follow-up. In the HSIL arm, two women never met for biopsy, and two women had only cytology follow-up. In total 20 women in the mRNA positive arm, 139 women in the ASCUS-LSIL arm, and 65 women in the HSIL arm had biopsies collected (Table 2).

**Table 2:** Status follow-up and biopsy outcomes.

	mRNA-positive arm	ASC_US- LSIL arm	HSIL arm
	N = 28	N = 452	N = 69
No follow-up	0	35	2
Back to screening at triage	8	199	
Incomplete follow-up	0	79	2
Histology			
Normal	5	24	2
CIN 1	6	30	2
CIN 2	1	17	6
CIN 3	8	66	53
Squamous cell ca.	0	2	1
Adenocarcinoma	0	0	1

Among women in the mRNA-positive arm, 32.2% (9/28) were CIN2+, respective 18.8% (85/452) in the ASC-US/LSIL-arm, and 88.4% (61/69) in the HSIL-arm. In total 146 women were diagnosed with CIN2+ in the ASC-US/LSIL- and HSIL-arms as practiced in the routine screening program (Table 2).

The overall screening program detection rate were 3.3% (95% CI: 2.8%-3.9%) for CIN2+, respective 2.8 (95% CI: 2.3%-3.3%) for CIN3+ (Table 3). By rescreening 28 samples (1.9%) HPV-mRNA positive of the 1 444 normal samples, and applying these findings to women with normal smear, not mRNA-tested, the overall program detection rate increased to 3.9% and 3.3% for CIN2+ and CIN3+ (Table 3), which constituted an estimated increase in screening program sensitivity of 16.4% (95% CI: 15.3–17.5) for CIN2+ and 17.3% (95% CI: 16.2–18.4) for CIN3+, respectively.

**Table 3:** Detection rates for CIN2+ and CIN3+ by status screening and estimated no. of CIN2+/CIN3+ cases among women with normal index cytology and no HPV-testing.

Outcome		Control arm*- normal smears– not mRNA tested	mRNA- positive arm	ASC_US-LSIL arm	HSIL arm	Total	Detection rate (95% CI)
		N = 2 401	N = 1 444	N = 452	N = 69	N = 4 366	
CIN 2+	As practiced			85	61	146	3.3 (2.8–3.9)
	+ rescreening	15*	9	85	61	170	3.9 (3.3–4.5)
CIN 3+	As practiced			52	55	123	2.8 (2.3–3.3)
	+ rescreening	13.3*	8	52	55	144.3	3.3 (2.8–3.8)

*Estimated no. of cases based on detection rate in the mRNA-positive arm.

During follow-up we diagnosed 23 cases of CIN2+ among 1 700 out 2 401women with normal smear and no mRNA testing at baseline. In this subset of women 19 CIN2/3 cases were tested at time for biopsy with a mRNA- or a DNA-test, among which 15 cases were positive for HPV-16, -18 or -45 in one (n = 8) or both tests (n = 7). Only one case was HPV-negative in both tests, while two cases were positive for HPV-31, and one case for HPV-33. There was 100% concordance between the mRNA- and the DNA-test results among the seven cases tested by both tests.

Norway has started, and will within 2021, implement national screening for cervical cancer with a primary DNA test for women 34 through 69 years of age, and continue cytology based screening for women 25 through 33 years. By restricting the analyses to women aged 23 to 33 years, the study population comprised 2770 women, with a HPV mRNA-positivity rate of 2.6%. The estimated increase in screening sensitivity for CIN2+ and CIN3+ in this subset of women were 21.3% (95% CI: 20.1–22.5) and 14.1% ((95% CI:13.1–15.1), respectively.

We diagnosed four cases of cervical cancer during follow-up after a positive screening cytology outcome (2 after ASCUS, 1 after HSIL and 1 after ASC-H) (Table 3). For three of these women index smear was first smear ever. Two women were diagnosed at age 23 and 24 before screening starts, and in one women at age 37when attending screening for the first time ever. All four cases were HPV positive (3 HPV-16, 1 HPV-31) and diagnosed in early stage 1A1.

## Discussion

By rescreening less than 2% of cytological normal samples that were positive for HPV E6/E7 mRNA types 16, 18 and 45, we achieved a significant increase in program detection rate for CIN3+ of 17–18%. Cervical cytology has a high rate of false negative samples. Low sensitivity for cytology in detection of CIN3 or invasive cervical cancer has been an issue for decades [23]. Case-series of women diagnosed with cervical cancer have revealed that younger women to a larger extend have a diagnosis of false negative smears during screening, while older women are under-screened or not participating in organized screening programs [21,24–26].

An improvement in diagnostic accuracy in cytology is one element of quality assessment in cervical cancer screening programs. While cytology represents morphological cellular changes, the detection of HPV mRNA expresses oncogenic molecular activity. Traditionally rescreening of a proportion of slides are considered an acceptable method for quality assessment if concordance of readings are high. However, this method is time consuming and tedious for cyto-technicians, and human errors are easily repeated (10). A molecular method is complementary to cytology in validation of diagnostic accuracy. It is therefore important that the molecular method target the HPV types that comprise the highest risk for progression to cervical cancer.

One way of expressing progression rate of precancerous lesions to cervical cancer is to study prevalence ratio of HPV types found in cancer tissue by types in tissue from CIN3. A ratio above 1.0 expresses an association, while an expression less than 1.0 weakens the potential oncogenic properties of the targeted HPV types. Worldwide, publications consistently report HPV 16, 18 and 45 with a prevalence ratio above 1.0, while most other HPV-types have prevalence ratios less than 0.4 [16,18,27]. The PreTect SEE test comprises the three HPV-types that are most important for progression of precancerous lesions into cancer among younger women, and detects oncogenic activity in false negative cytology samples.

Transient infections of many HPV-types are prevalent in the female genital tract. Most women have normal smear readings from cervix uteri in situations of transient infections [28,29]. In order to increase accuracy of cytology as method in cervical screening, it is important that a complementary HPV validation-test target oncogenic activity among major HPV-types in the etiology of cervical cancer. An HPV-DNA-test covering many types will detect too many transient infections leading to a higher rescreening rate, followed by more referrals to colposcopy in situations of HPV-infections with low potential of progression of the normal cell to CIN2/3 [30], and little potential, if any at all, of progression into cancer. This is crucial in surveillance of younger women where the majority of CIN2 cases regress to normal cells within few years [31–33].

The strength of our study is the population-based study design in a country having had a well-organized screening program for over 20 years. We excluded women with a history of high-grade cytology and/or CIN1+ that would represent persistent HPV infections, thus leaving a subset of low-risk women being exposed or not exposed to a probable incident HPV infection with oncogenic expression.

Our study reflects the “real life” situation, the way women participate in the screening program, and how women and their doctors adhere to the recommended follow-up algorithms. We tried to avoid observation and attrition bias by comparing follow-up outcomes within one screening interval. The follow-up of women with normal cytology and no mRNA testing (N = 1701) revealed 23 incident cases of CIN2/3, among which 15 out of 19 tested positive for HPV 16, 18 and 45. This indicates that the estimates for increase in screening sensitivity by rescreening all women with normal cytology with a positive HPV mRNA test may be an underestimate of what is possible to achieve in a real life among women less than 40 years.

According to annual reports from the NCCSP 1058 of 2712 (39%) cervical cancers were diagnosed among women less than 40 years in Norway over the 10-year period 2007 through 2016 (Table 1). Among women less than 40 years of age, 603 women had at least one valid smear prior start of examinations leading to a cancer diagnoses. In this subset, 344 (57%) women had a false normal smear at the most recent visit within 4 years of cancer. Norway will continue with cytology based screening in women aged 25 through 33 years after implementation of primary HPV DNA-based screening. As most cervical cancers develop over decades, and in order to diagnose the more aggressive HPV-types, it will be important to validate normal smears with a molecular method in the younger age groups, at a time point where the lesions have not penetrated the basal membrane of the cervix uteri. It was in this subset of women we increased the detection rate by 14–15% for CIN3 when applying a mRNA test as validation of presumptive normal smears. Our sample size is not large enough for discovering any cases of cervical cancer in the group of women having a normal smear and no mRNA testing at baseline, but large enough for a valid confirmation of estimated versus observed cases of CIN3.

In 2011 the costs of the NCCSP were NOK 730 millions when approximately 3500 CIN2+ were diagnosed [34]. If we assume that the costs for the NCCSP has remained at this level for the most recent years an average cost for a CIN2+ in NCCSP (n = 6000) [35] were NOK 120 000 (USD 15000) in 2016. If we apply the results from the present study to rescreening of the 100 000 women with normal screens aged 23–39 years in Norway (mRNA test = NOK 250 all costs included), rescreening rate of 2% (a cytology examination reimbursed with NOK 70), more than 540 (95% CI: 500–590) new cases of CIN2+ may be detected. An average cost for an additional case of CIN2+ with rescreening of assumed normal smears will be NOK 46666 (95% CI: NOK 42610–50280). The women rescreened are in the system, and they do not need reminders if no show-up in subsequent screening rounds. Rescreening of normal smears with a mRNA test may be cost-effective in case-finding of CIN2+. However, these results have to be confirmed in more detailed cost-effectiveness analyses of rescreening presumable normal smears with a 3-type mRNA test from results in prospective ongoing studies.

In order to prevent cervical cancer with screening in women less than 40 years, high quality diagnosis and treatment of precancerous lesions before age 34 will be important for many years. In Norway, we have to wait until 2037 before the fully vaccinated cohorts reach 40 years.

## Conclusions

By testing all women less than 40 years of age with normal cytology with a specific 3-type HPV mRNA test, we achieved a significant (17–18%) increase in screening program sensitivity for CIN3. The 3-type HPV mRNA is a high throughput assay, representing similar workload as any HPV DNA test commonly used in primary screening. The volume of rescreened smears (1.9%) represent a low workload. In addition, the study adds quality to educating the screeners by rescreening presumably false negative Pap-smears.
